# Durability of and role of AKT in FGF7p urothelial protection against cyclophosphamide

**DOI:** 10.14814/phy2.15358

**Published:** 2022-06-24

**Authors:** Sridhar Tatarao Narla, Lori Rice, David Ostrov, Daniel Scott Bushnell, Joanne Lindsey Duara, Carlton Matthew Bates

**Affiliations:** ^1^ Division of Nephrology Department of Pediatrics University of Pittsburgh School of Medicine Pittsburgh Pennsylvania USA; ^2^ Department of Radiation Oncology College of Medicine University of Florida Gainesville Florida USA; ^3^ Department of Pathology, Immunology, and Laboratory Medicine University of Florida College of Medicine Gainesville Florida USA; ^4^ Division of Neonatology, Department of Pediatrics University of Pittsburgh School of Medicine Pittsburgh Pennsylvania USA; ^5^ Division of Nephrology UPMC Children's Hospital of Pittsburgh Pittsburgh Pennsylvania USA

**Keywords:** AKT, bladder urothelium, cyclophosphamide, FGF7p, fibroblast growth factor 7 peptide

## Abstract

We previously identified a peptide derived from human fibroblast growth factor 7 (FGF7p) that blocks urothelial apoptosis similar to full‐length FGF7, although effects of FGF7p on urothelial repair are unknown. Also, while urothelial AKT activation downstream of FGF7p correlated with the anti‐apoptotic effects, we have not directly interrogated the role of AKT in mediating the cytoprotection. Our goal was to assess effects of FGF7p on urothelial repair and the role of AKT signaling in mediating the cytoprotective effects of FGF7p. We performed hematoxylin and eosin (H&E), TUNEL, and/or immunofluorescence (IF) staining for various markers in FGF7p‐treated mice 28 days after giving cyclophosphamide or after co‐administering a systemic AKT antagonist with FGF7p 24 h after cyclophosphamide. Vehicle‐treated and injured mice had hyperplastic urothelium, incomplete return of mature superficial cell markers, ongoing proliferation, and continued presence of basal progenitor markers 28 days after injury; conversely, FGF7p‐treated mice had normal numbers of urothelial cell layers, nearly complete return of superficial cell markers, limited proliferation and fewer basal progenitor cells 28 days post‐injury. Vehicle‐treated mice also had ectopic lumenal basal progenitor cell markers, while FGF7p had none 28 days after cyclophosphamide. Co‐administration of an AKT inhibitor largely abrogated FGF7p‐driven AKT activation and cytoprotection in urothelium 24 h after injury. Thus, FGF7p drives faster and higher fidelity urothelial repair by limiting apoptotic injury via AKT signaling, similar to full‐length FGF7. Finally, FGF7p is much less expensive to synthesize and has a longer shelf life and higher purity than FGF7.

## Graphical abstract

Fibroblast growth factor 7 peptide mimetic (FGF7p) administration leads to faster and higher fidelity repair of bladder urothelium 28 days after cyclophosphamide injury. The FGF7p‐driven urothelial cytoprotection 24 hours after cyclophosphamide appears to be dependent on AKT signaling in that an AKT inhibitor blocks the protective effects. Thus, FGF7p mitigates bladder urothelial injury and drives better repair after cyclophosphamide similar to full length FGF7.

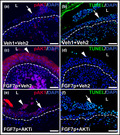

## 1 INTRODUCTION

Cyclophosphamide, an alkylating agent used to treat many types of cancer and non‐oncological disease, often leads to urothelial bladder injury (Haldar et al., 2014; Korkmaz et al., 2007; Nicol, 2002). Acrolein, a metabolite of cyclophosphamide is highly bladder toxic, can cause significant urothelial cell apoptosis and necrosis (Narla, Bushnell, Schaefer, Nouraie, & Bates, 2020). Acute injury ranges from minor lower urinary tract symptoms to life‐threatening hemorrhagic cystitis. Long‐term complications can include bladder fibrosis and urothelial cancer that can occur much later in life (Baker et al., 1987; Kaldor et al., 1995; Shirai, 1993; Travis et al., 1995; Vlaovic & Jewett, 1999). Current treatments to prevent cyclophosphamide bladder injury such as hydration (to dilute acrolein) or sodium‐2‐mercaptoethanesulfonate (to bind acrolein) have variable acute outcomes and have not prevented long term complications such as cancer (Korkmaz et al., 2007; Matz & Hsieh, 2017; Moghe et al., 2015).

Another potential therapeutic agent to block cyclophosphamide/acrolein bladder toxicity is fibroblast growth factor 7 (FGF7). Over two decades ago, a study showed that systemic infusion of recombinant human FGF7 in rats prior to cyclophosphamide led to better urothelial histological outcomes, although it was unclear if the benefits were through cytoprotection or enhanced repair (Ulich et al., 1997). More recently, our group showed that administration of FGF7 before giving cyclophosphamide virtually completely blocked urothelial cell apoptosis 24 h after injury leading to faster and higher fidelity of repair in mice (Narla, Bushnell, Schaefer, Nouraie, & Bates, 2020). We found that FGF7 activated it's receptor, the epithelial isoform of fibroblast growth factor receptor 2 (FGFR2IIIb), in urothelium, followed by phosphorylation (activation) of AKT; AKT activation correlated temporally with the anti‐apoptotic effects of FGF7. We later published that chemical inhibition of AKT blocked the cytoprotective effects of FGF7 from cyclophosphamide and that a direct AKT agonist alone was sufficient to drive urothelial cytoprotection against cyclophosphamide (Narla et al., 2021).

While full‐length FGF7 has promise to prevent cyclophosphamide injury in patients, concerns include that it is expensive to produce using recombinant bacterial means, leading to high consumer costs. We previously reported a peptide mimetic derived from FGF2 that could be directly synthesized (reducing production costs) and that worked as effectively as full‐length recombinant human FGF2 in blocking intestinal injury from radiation (Zhang et al., 2010). More recently we identified and directly synthesized a small peptide fragment from FGF7, called FGF7 peptide (FGF7p), analogous to the FGF2 mimetic (Narla et al., 2022). Moreover, systemic FGF7p infusion activated AKT in mouse urothelium and was able to block cyclophosphamide‐induced apoptosis 24 h after injury, similar to full‐length FGF7. The present study examined whether FGF7p administration led to faster and higher fidelity of repair 28 days after cyclophosphamide. We also directly interrogated the role of AKT signaling downstream of FGF7p by co‐administering the mimetic and a chemical AKT antagonist to determine if the latter blocked the protective effects of the peptide.

## 2 MATERIALS AND METHODS

### 2.1 Mice

We used 2–3 months old female FVB/NJ mice for all assays (The Jackson Laboratory). All of the mouse assays were approved by the University of Pittsburgh Institutional Animal Care and Use Committee in compliance with guidelines from the Association for Assessment and Accreditation of Laboratory Animal Care.

### 2.2 Drugs/chemicals given to mice

We gave mice two 20 mg/kg subcutaneous (SQ) injections of FGF7p (Narla et al., 2022) dissolved in 2.5% dimethyl sulfoxide (DMSO) (Sigma‐Aldrich, Cat# D2438) or 2.5% DMSO alone (Vehicle 1), one dose at 72 h and the other dose at 48 h before cyclophosphamide. To inhibit AKT signaling, we administered 40 mg/kg intraperitoneal (IP) injections of LY294002 (Selleckchem, Cat# S1105), a known AKT inhibitor (AKTi) dissolved in 2% DMSO (Sigma‐Aldrich, Cat# D2438) or 2% DMSO alone (Vehicle 2) concurrently with the initial dose of FGF7p and then every 12 h until termination of the experiments. To induce bladder injury with FGF7p and/or the AKTi, we administered 150 mg/kg IP injections of cyclophosphamide (Sigma‐Aldrich, Cat# C7397) dissolved in PBS. For experiments with FGF7p alone, we harvested bladders 28 days after cyclophosphamide. For experiments with FGF7p and the AKT inhibitor, we harvested bladders 24 h after cyclophosphamide.

### 2.3 Histology, immunofluorescence, and TUNEL assays

We isolated and fixed bladders in 4% paraformaldehyde (PFA), processed and embedded tissues in paraffin, and serially sectioned bladders at 6 μm. For general histology, we stained with hematoxylin and eosin (H&E). For immunofluorescence (IF), we dewaxed the paraffin‐embedded sections and subjected them to antigen retrieval in a pressure cooker for 15 min in Tris‐EDTA pH 9.0 buffer. We then blocked with normal donkey serum for 1 h at room temperature (RT). We then incubated sections overnight at 4°C with the following primary antibodies: Anti‐Keratin 20 (KRT20, mature superficial cell marker) at 1:50 (Agilent Technologies, Cat# M7019, RRID:AB_2133718), Keratin 5 (KRT5, basal and intermediate cell subset marker) at 1:200 (BioLegend, Cat# 905901, RRID:AB_2565054), Keratin 14 (KRT14, basal cell progenitor marker) at 1:200 (BioLegend, Cat# 905301, RRID:AB_2565048), Uroplakin 3a (UPK3, superficial cell and intermediate cell subset marker) at 1:200 (Santa Cruz Biotechnology, Cat# sc‐33,570, RRID:AB_2213486), pAKT at 1:100 (Cell Signaling Technology, Cat# 4060, RRID:AB_2315049), and Ki‐67 (Ki67, marker of proliferation) at 1:200 (R&D Systems Cat# AF7649, RRID:AB_2687500). After washing in PBS, we incubated slides with the following secondary antibodies: Alexa Fluor 594 (Thermo Fisher, Cat# A‐21207, RRID:AB_141637), Alexa Fluor 488 (Thermo Fisher, Cat# A‐21202, RRID:AB_141607) and/or Alexa Fluor 647 (Jackson ImmunoResearch Labs, West Grove, PA Cat# 711–605‐152, RRID:AB_2,492,288) all at 1:500 for 2 h at RT, followed by washes. We stained nuclei with 4′6′‐diamidino‐2‐phenylindole (DAPI) (Sigma Aldrich, Cat# D1306). To assay for apoptosis, we performed TUNEL assays with ApopTag Plus in situ Apoptosis Fluorescein Detection kit (EMD Millipore, Cat# S7111) according to the manufacturer's protocol. We performed staining for all conditions in two planes containing entire rings of urothelium per bladder with three mice of each treatment group per timepoint (thus *N* = 6 for each timepoint). We imaged slides with a Leica DM2500 microscope (Leica Microsystems) or a Zeiss LSM 710 confocal microscope (Carl Zeiss). We obtained images from both microscopes at room temperature with a 20× objective at the same settings for vehicle and treatment groups, including the same exposure time. Any subsequent adjustments to brightness and/or contrast on captured images were identical for vehicle and treatment groups.

We assessed total urothelial proliferation rates by counting Ki67^+^/ all DAPI^+^ urothelial cells, KRT14^+^ cell proliferation rates by counting Ki67^+^ and KRT14^+^/ all DAPI^+^ urothelial cells and KRT14^−^ urothelial cell proliferation rats by counting Ki67^+^ and KRT14^−^/ all DAPI^+^ urothelial cells 28 days after injury. We also calculated the percentage of all KRT14^+^ cells 28 days after injury by counting all KRT14^+^ cells/all DAPI^+^ urothelial cells. Finally, we assessed the mean number of foci of ectopic lumenal KRT14 staining 28 days after injury. For all assays, we performed counts in two planes containing entire rings of urothelium per bladder with three mice of each treatment group per timepoint (thus *N* = 6 for each timepoint). The personnel that performed counts were blinded to the treatment conditions of the mice.

### 2.4 Statistical analysis

Quantitative data are represented as means ± SEM. To compare urothelial cell proliferation rates (Ki67^+^/DAPI^+^, Ki67^+^/KRT14^+^ and Ki67^+^/KRT14^−^ urothelial cells), we performed an analysis variance and a Bonferroni post hoc test. To compare percentages of KRT14^+^ cells and mean numbers of ectopic KRT14 foci, we performed Students *t*‐tests. We used a two‐sided hypothesis test for all analyses.

## 3 RESULTS

### 3.1 FGF7p treatment appears to lead to faster repair of urothelium 28 days after cyclophosphamide

**FIGURE 1:**
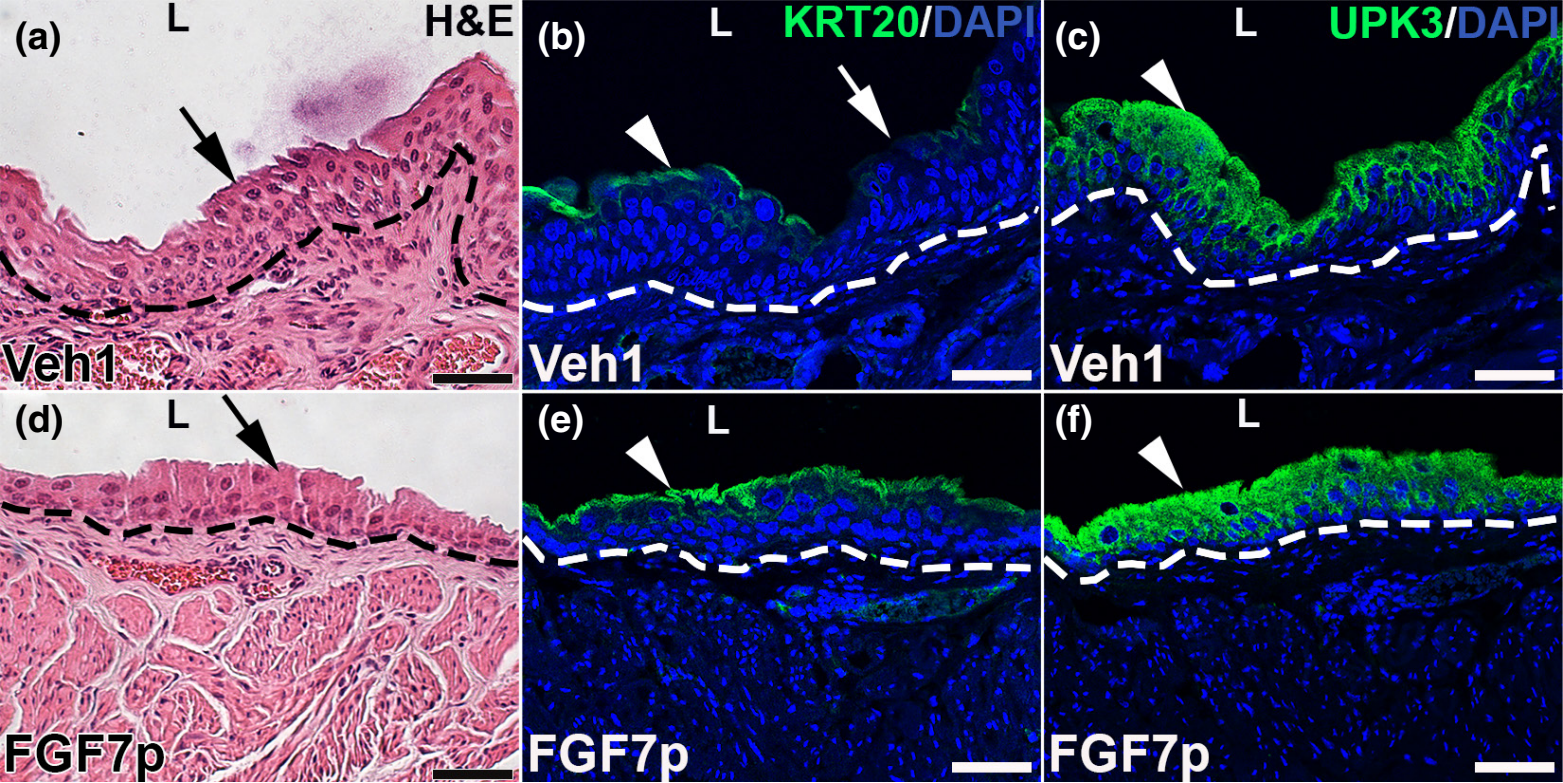
Representative images of urothelial repair in Vehicle 1 (Veh1) and FGF7p‐treated mice 28 days after cyclophosphamide. (a, d) H&E images reveal that the Vehicle 1‐treated mice (a) have hyperplastic urothelium with 6–7 cell layers (arrow), while the FGF7p‐treated mice (d) have 3–4 cell layers similar to uninjured urothelium (arrow). (b, e) IF for KRT20 (green) shows that Vehicle 1‐treated mice (b) have some regions with staining (arrowhead) but others with absent/attenuated staining (arrow) suggesting incomplete return of mature superficial cells, while FGF7p‐treated mice (e) have nearly continuous staining (arrowhead), consistent with almost complete return of superficial cells. (c, f) If for UPK3 (green) shows that both Vehicle 1‐treated (c) and FGF7p‐treated (f) mice have strong signal (arrowheads), consistent with return of intermediate cell subsets and/or immature superficial cells. Blue (b–c, e–f) = DAPI. L (a–f) = Lumen. Dashed line (a–f) = urothelial‐stromal boundary. Scale bars (a–f) = 50 μm.

We previously found that systemic administration of FGF7p prior to cyclophosphamide significantly blocked urothelial apoptosis 24 h after injury, similar to full‐length FGF7 (Narla et al., 2022). We now examined whether the early cytoprotection led to faster repair of urothelium 28 days after injury, as was true with full‐length FGF7 (Narla, Bushnell, Schaefer, Nouraie, & Bates, 2020). H&E staining in mice that were pre‐treated with Vehicle 1 and then injured showed bladders with 6–7 urothelial cell layers in most of the sections imaged, consistent with hyperplasia and ongoing bladder regeneration (Figure 1). In contrast, those given FGF7p had 3–4 cell layers within 28 days of injury, which is similar to quiescent and uninjured urothelium (Figure 1). Immunofluorescence (IF) for KRT20 showed that Vehicle 1‐pretreated mice had many regions with no signal consistent with only partial return of mature superficial cells, while FGF7p‐pretreated mice had almost continuous staining suggesting nearly complete return of mature superficial cells (Figure 1). IF for UPK3 showed relatively similar staining in both Vehicle 1 and FGF7p‐ pretreated mice, consistent with the return of intermediate cell subsets and/or immature superficial cells in each group (Figure 1). Thus, as was true with full‐length FGF7 (Narla, Bushnell, Schaefer, Nouraie, & Bates, 2020), pretreatment with FGF7p appears to drive faster repair of urothelium 28 days after cyclophosphamide.

**FIGURE 2:**
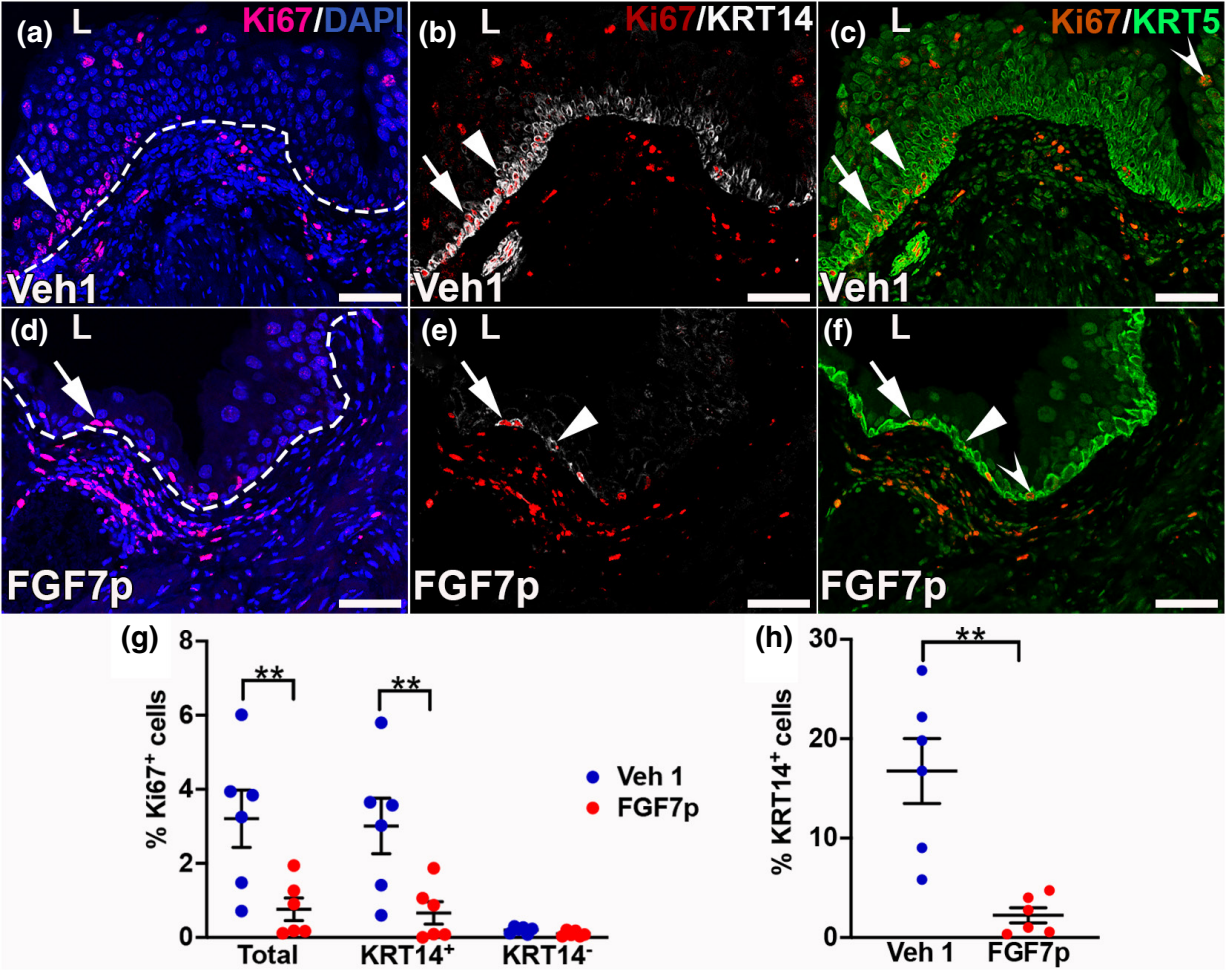
Representative images showing many higher numbers of proliferating and KRT14^+^ urothelial cells in Vehicle 1 (Veh1)‐treated versus FGF7p‐treate mice 28 days after cyclophosphamide. (a–f) Triple label IF for Ki67 (red), KRT14 (white), and KRT5 (green) in Vehicle 1‐treated (a–c) and FGF7p‐treated (d–f) mice. (a, d) IF for Ki67 with DAPI (blue) shows that the Vehicle 1‐treated mouse (a) has many proliferating urothelial cells, with the majority found in basal layers (arrow) while the FGF7p‐treated mouse (d) has significantly fewer proliferating urothelial cells that are also primarily in basal layers (arrow). (b, e) Merged Ki67 and KRT14 staining shows that both the vehicle 1‐treated mouse (b) and the FGF7p‐treated mouse (e) have many KRT14^+^ basal cells that are proliferating (arrows) and other KRT14^+^ cells that are not proliferating (arrowheads), although the Vehicle 1‐treated has many more of each than the FGF7p‐treated mouse. (c, f) Merged Ki67 and KRT5 staining shows that in the both the Vehicle 1‐treated mouse (c) and the FGF7p‐treated mouse, all of the KRT14^+^ cells that are proliferating (arrows) or not proliferating (arrowheads) are KRT5^+^, although some of the proliferating KRT5^+^ cells are KRT14^−^ (concave arrowheads). L (a–f) = lumen. Dashed line (a, d) = urothelial‐stromal boundary. Scale bars (a–f) = 50 μm. (g) Graph showing that the mean percentage of total Ki67^+^ proliferating urothelial cells and mean percentage of KRT14^+^ proliferating urothelial cells are higher in the Vehicle 1‐treated mice than FGF7p‐treated mice 28 days after injury; there are no differences in percentages of KRT14‐ proliferating urothelial cells between the two groups. *N* = 6 (3 bladders with two planes assessed per mouse). **p < 0.01. (h) Graph showing that the mean percentage of KRT14^+^ cells is higher in the Vehicle 1‐treated mice than the FGF7p‐treated mice 28 days after injury. *N* = 6 (three bladders with two planes assessed per mouse). **p < 0.01.

We next assessed the degree of ongoing regeneration in mice pretreated with Vehicle 1 or FGF7p 28 days after injury. To that end we performed triple label immunofluorescence for Ki67 (marker of proliferation), KRT14 (basal cell progenitor marker), and KRT5 (marker of all basal cells and intermediate cell subsets that are typically UPK3 negative). Vehicle 1‐treated and injured mice appeared to have more Ki67^+^ proliferating urothelial cells than FGF7p‐treated and injured mice (Figure 2). Formal quantification confirmed higher mean total urothelial cell proliferation rates in injured mice given vehicle 1 than in those treated with FGF7p (Vehicle 1: 3.2% ± 0.78%, FGF7p: 0.76% ± 0.030%, *p* < 0.01) (Figure 2). We also noted many unbroken stretches of KRT14^+^ basal cells in the bladders in Vehicle 1‐treated mice, consistent with ongoing regeneration, as opposed to more rare KRT14^+^ cells in FGF7p‐treated mice, largely reminiscent of quiescent bladders (Figure 2). Formal quantification confirmed a much higher mean percentage of KRT14^+^ cells in Vehicle 1‐treated mice than FGF7p‐treated mice (Vehicle 1: 17% ± 3.3%, FGF7p: 2.2% ± 0.76%, *p* < 0.01) (Figure 2). Merged Ki67 and KRT14 staining appeared to show that most of the proliferating cells were also KRT14^+^ in both groups (Figure 2). Formal quantification confirmed that most of the Ki67^+^ urothelial cells in both groups were KRT14^+^, but that the mean percentage of proliferating KRT14^+^ cells was much higher in the Vehicle 1‐treated than the FGF7p treated mice (Vehicle 1: 3.0% ± 0.75%, FGF7p: 0.66% ± 0.30%, *p* < 0.01) (Figure 2). Mean percentages of proliferating KRT14^−^ urothelial cells (either KRT5^+^ or KRT5^−^) were relatively low and not significantly different between the two groups (Vehicle 1: 0.20% ± 0.036%, FGF7p: 0.098% ± 0.030%, *p* > 0.05) (Figure 2). Taken together, the FGF7p‐treated group had much less proliferation and greater regression of KRT14^+^ basal progenitor cells compared to Vehicle 1‐treated mice, consistent with earlier resolution of regeneration in the former compared to the latter. Again, these findings mimic what we observed with full‐length FGF7 pretreatment versus vehicle after cyclophosphamide injury (Narla, Bushnell, Schaefer, Nouraie, & Bates, 2020).

### 3.2 FGF7p treatment appears to lead to higher fidelity repair of urothelium 28 days after cyclophosphamide

**FIGURE 3:**
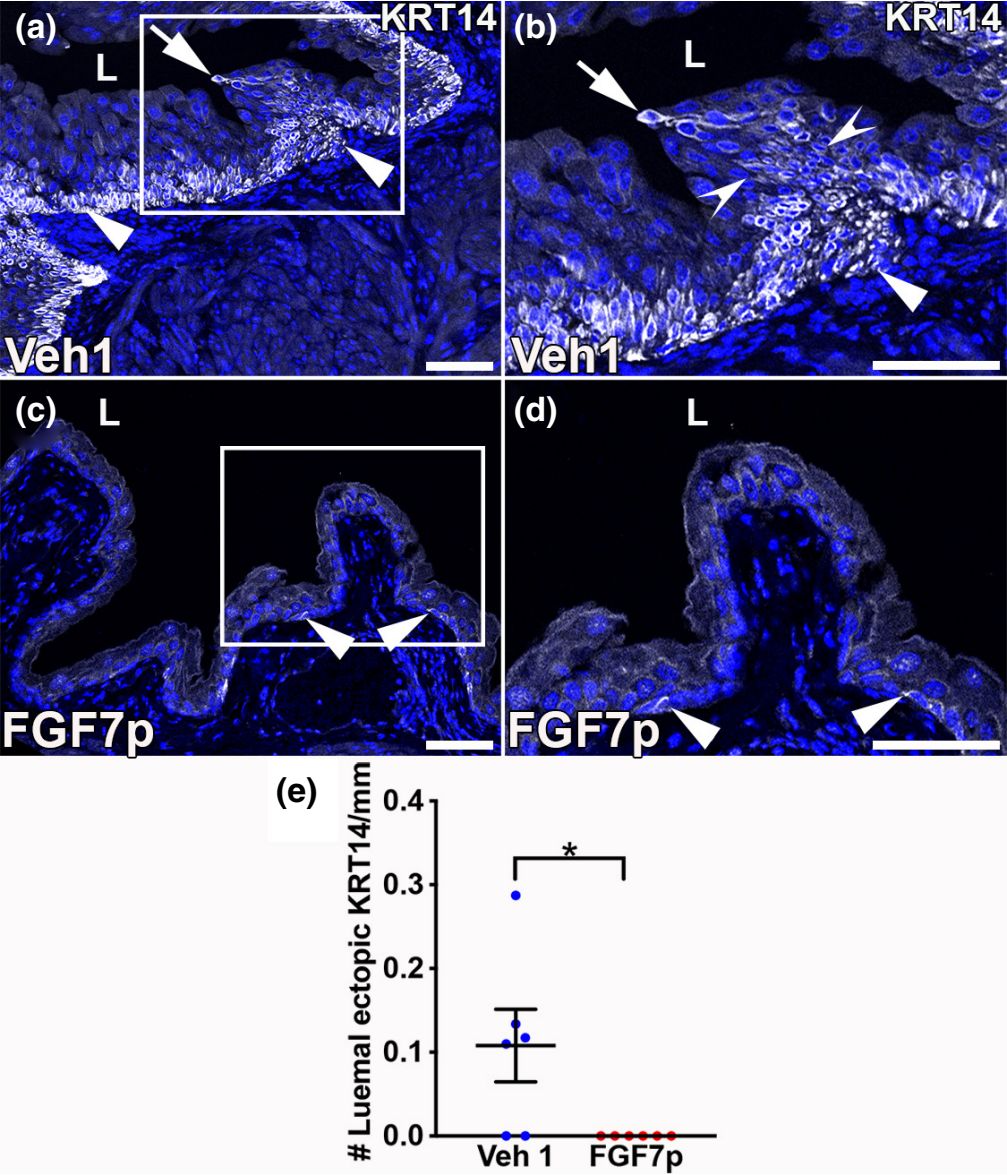
Representative images showing how FGF7p‐treated mice have limited KRT14 staining and no ectopic lumenal foci compared to Vehicle 1 (Veh1)‐treated mice 28 days after cyclophosphamide. (a–d) IF for KRT14 (white) shows that the Vehicle 1‐treated and injured mouse (a) has widespread numbers of basal KRT14^+^ cells (arrowheads) and an ectopic lumenal KRT14^+^ cell (arrow). A higher power magnification of the boxed region in panel a re‐demonstrates the ectopic KRT14^+^ cell (arrow) arising from a column of KRT14^+^ cells (between concave arrowheads) that are attached to basal KRT14^+^ cells (arrowhead) in the Vehicle 1‐treated mouse. A lower power magnification (c) and higher power magnification from the boxed region in panel c (d) show that the FGF7p‐treated and injured mouse has many fewer basal KRT14^+^ cells (arrowheads) and no ectopic lumenal foci compared to the Vehicle 1‐treated mouse. Blue (a–d) = DAPI. L (a–d) = lumen. Scale bars (a–d) = 50 μm. (e) Graph showing that the mean number of ectopic lumenal KRT14^+^ foci is higher in the Vehicle 1‐treated mice than FGF7p‐treated mice 28 days after injury. *N* = 6 (3 bladders with 2 planes assessed per mouse). *p < 0.05.

We have previously noted that a single dose of cyclophosphamide can lead to evidence of maladaptive repair in wild‐type mice manifested as ectopic lumenal expression of KRT14^+^ cells (Narla, Bushnell, Schaefer, Nouraie, Tometich, et al., 2020). We then tested whether FGF7p treatment would prevent cyclophosphamide‐induced ectopic lumenal expression of KRT14. As expected, we observed ectopic lumenal KRT14^+^ foci in Vehicle 1‐treated mice 28 days after injury, typically emanating from columns of KRT14^+^ cells attached to KRT14^+^ basal cells (Figure 3). In contrast, we never observed any KRT14^+^ cells at the lumenal surface in mice pretreated with FGF7p 28 days after cyclophosphamide (Figure 3). Formal quantification confirmed that compared to Vehicle 1‐treated mice, FGF7p‐treated mice had lower mean numbers of ectopic KRT14^+^ foci per mm lumenal surface (Vehicle 1: 0.11 ± 0.043, FGF7p: 0 ± 0, *p* < 0.05). Thus, FGF7p appears to drive higher fidelity repair of urothelium 28 days after cyclophosphamide injury.

### 3.3 FGF7p appears to block urothelial apoptosis via AKT signaling

**FIGURE 4:**
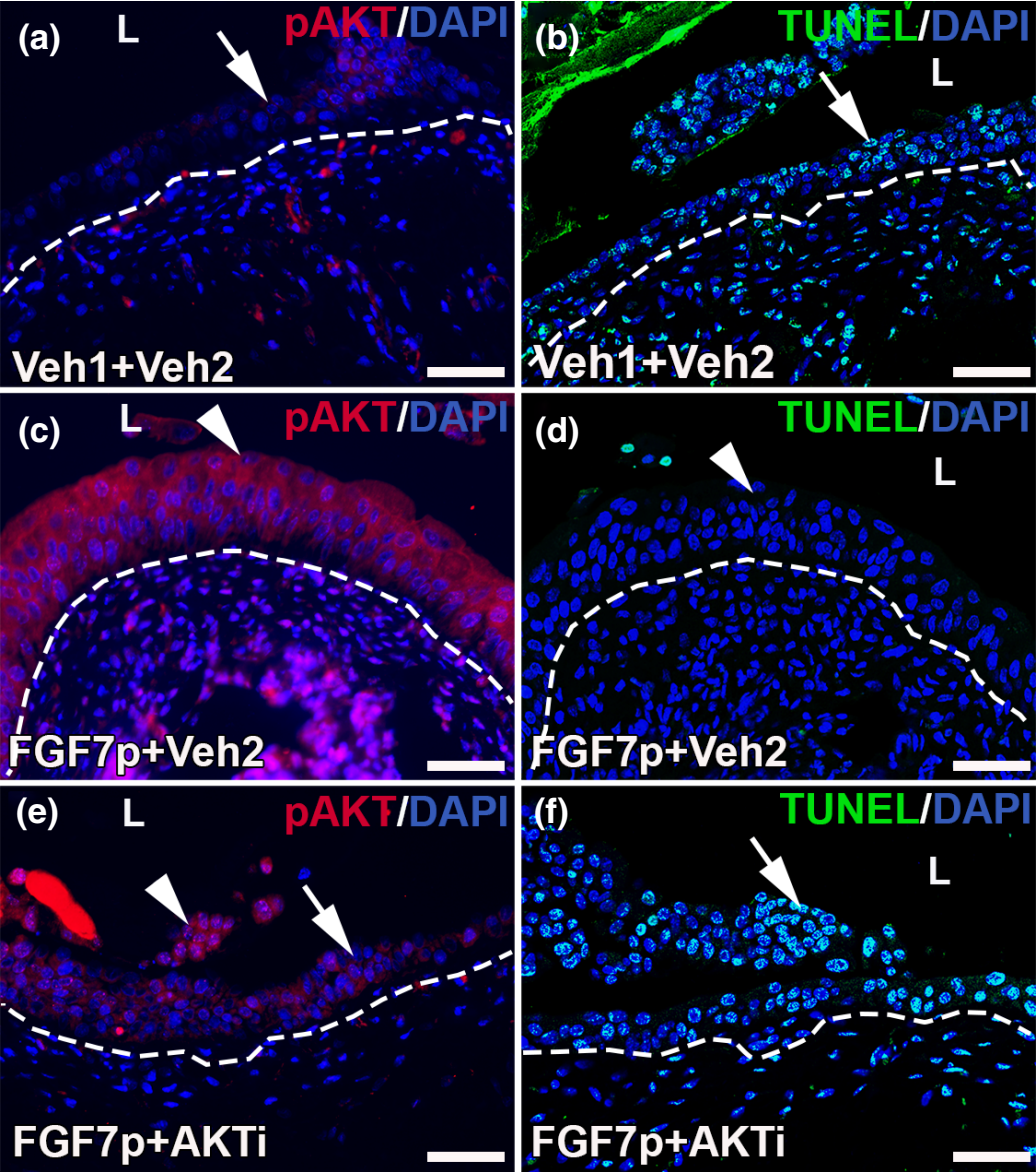
Representative images showing that the AKT inhibitor (AKTi) blocks FGF7p‐indcued urothelial pAKT expression and cytoprotection 24 h after cyclophosphamide. (a, c, e) IF for pAKT (red). (b, d, f) TUNEL staining (green). (a–b) injured mice treated with Vehicle 1 (Veh1) and Vehicle 2 (Veh2) have almost no urothelial pAKT staining (a, arrow) and have widespread urothelial TUNEL staining (B, arrow). (c–d) Injured mice treated with FGF7p and Vehicle 2 have significant induction of urothelial pAKT staining (c, arrowhead) and virtually no TUNEL^+^ apoptotic urothelial cells (d, arrowhead). (e–f) injured mice treated with FGF7p and the AKT inhibitor have few patches of urothelial pAKT staining (e, arrowhead) among most of the urothelium that has no signal (e, arrow) and have breakthrough TUNEL^+^ apoptosis throughout most of the urothelium (f, arrow). Blue (a–f) = DAPI. L (a–f) = lumen. Dashed line (a–f) = urothelial‐stromal boundary. Scale bars (a–f) = 50 μm.

We next interrogated the role of AKT signaling downstream of FGF7p in mediating the FGF7p‐driven cytoprotective effects. We previously reported that FGF7p treatment led to phosphorylation of AKT in the urothelium, which correlated with the anti‐apoptotic effects of the peptide (Narla et al., 2022). We also previously found that full‐length FGF7 activated AKT in the urothelium and that co‐administration of an AKT antagonist with FGF7 blunted phosphorylation of AKT, leading to breakthrough urothelial apoptosis after cyclophosphamide (Narla et al., 2021; Narla, Bushnell, Schaefer, Nouraie, & Bates, 2020). To directly interrogate the role of AKT in mediating cytoprotection downstream of FGF7p, we co‐administered FGF7p (or Vehicle 1) and the AKT inhibitor LY294002 (Azaro et al., 2015) (or Vehicle 2) in cyclophosphamide‐injured mice and harvested 24 h after injury. Mice given Vehicle 1 and Vehicle 2 had virtually no urothelial pAKT expression and had significant urothelial apoptosis 24 h after cyclophosphamide (Figure 4), similar to what we previously found in injured mice treated with Vehicle 1 only (Narla et al., 2022). Injured mice treated with Vehicle 1 and the AKTi had similar pAKT and TUNEL urothelial staining patterns as the Vehicle 1 and Vehicle 2‐treated mice (not shown). In contrast, injured mice given FGF7p and Vehicle 2 had significant increases in urothelial pAKT staining and suppression of urothelial apoptosis 24 h after injury (Figure 4), similar to what we observed with FGF7p treatment alone (Narla et al., 2022). FGF7p and AKTi co‐treated and injured mice with almost complete suppression of FGF7‐induced urothelial pAKT staining and had significant urothelial apoptosis, reminiscent of injured mice treated with Vehicle 1 and Vehicle 2 (Figure 4).

**FIGURE 5:**
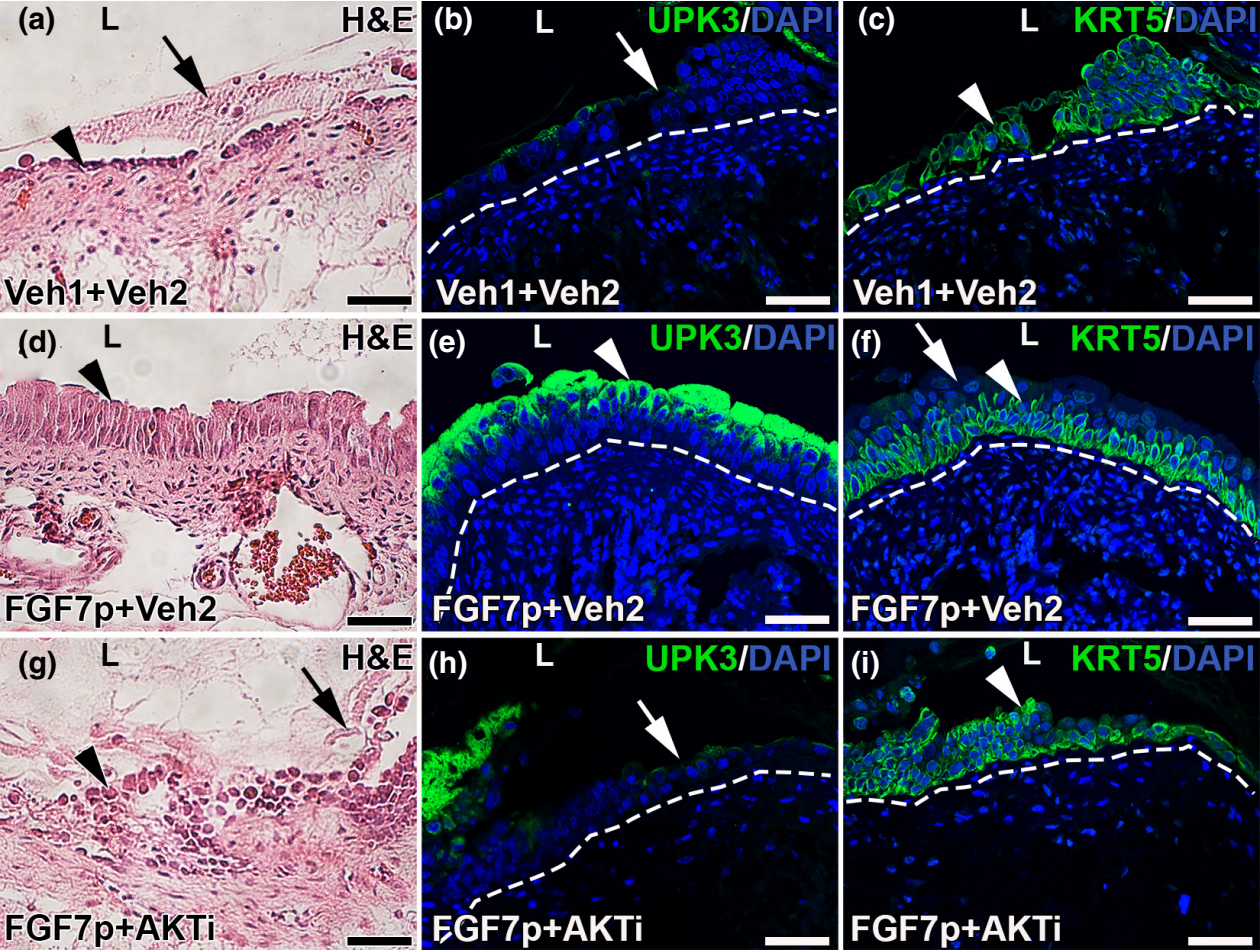
Representative images showing how the AKT inhibitor (AKTi) blocks FGF7p‐indcued preservation of urothelium 24 h after cyclophosphamide. (a, d, g) H&E staining. (b, e, h) IF for UPK3 (green). (c, f, i) IF for KRT5 (green). (a–c) Injured mice treated with Vehicle 1 (Veh1) and Vehicle 2 (Veh2) have large areas of urothelial sloughing and debris (a, arrow) with injured‐appearing remnant urothelium (a, arrowhead) by H&E staining, almost complete loss of UPK3^+^ cells (B, arrow), and injured/dissociating KRT5^+^ cells (C, arrowhead). (d–f) Injured mice treated with FGF7p and Vehicle 2 have largely preserved urothelium by H&E staining (D, arrowhead), relatively intact UPK3^+^ intermediate cell layers (E, arrowhead), and relatively undamaged KRT5^+^ intermediate and basal cell layers (F, arrowhead) with preserved KRT5^−^ staining in cells at the lumenal surface (F, arrow) that represent the UPK3^+^ cells. (g–i) injured mice treated with FGF7p and the AKT inhibitor have large regions of urothelial cell debris and sloughing (G, arrow) with unhealthy‐appearing remnant urothelium (G, arrowhead) by H&E staining, almost complete loss of UPK3^+^ cells (H, arrow) and injured/dissociating KRT5^+^ cells (I, arrowhead), all reminiscent of the Veh1 and Veh2‐treated mice. Blue (B, C, E, F, H, I) = DAPI. L (A‐I) = lumen. Dashed line (B, C, E, F, H, I) = urothelial‐stromal boundary. Scale bars (A‐I) = 50 μm.

We next assessed H&E staining and urothelial cell‐specific markers in the various treatment groups 24 h after cyclophosphamide. As expected, the mice treated with Vehicle 1 and Vehicle 2 had significant urothelial injury on H&E staining characterized by urothelial cell sloughing and damaged remnant urothelium (Figure 5), similar to what we previously reported in injured mice treated with Vehicle 1 only (Narla et al., 2022). IF for KRT20 showed complete loss of staining, consistent with loss of mature superficial cells in the Vehicle 1 + Vehicle 2 group (not shown). IF for UPK3 also showed almost a complete loss of signal, consistent with loss of UPK3^+^ intermediate cells in the Vehicle 1 + Vehicle 2‐treated mice and IF for KRT5 showed injured and dissociating KRT5^+^ intermediate cell subsets and basal cells (Figure 5). We saw similar disrupted urothelial staining patterns by H&E and IF in mice given Vehicle 1 and AKTi (not shown). As expected, injured mice treated with FGF7p and Vehicle 2 had relatively intact urothelium by H&E staining (Figure 5). IF for KRT20 showed complete loss of staining, consistent with loss of mature superficial cells in the FGF7p + Vehicle 2 treated group (not shown), which is in alignment with our previous studies showing that both full‐length FGF7 and FGF7p did not prevent superficial cell necrosis after cyclophosphamide (Narla et al., 2022; Narla, Bushnell, Schaefer, Nouraie, & Bates, 2020). IF for UPK3 and KRT5 showed robust and intact staining in the injured FGF7p + Vehicle 2‐treated mice, consistent with the preservation of both intermediate cell types and basal cells (Figure 5). Injured mice treated with FGF7p and the AKTi had staining patterns that largely resembled the vehicle 1 and vehicle 2‐treated mice with H&E staining showing urothelial cell sloughing and damaged remnant cells, almost complete loss of UPK3^+^ cells and significant injury to the remaining KRT5^+^ cells (Figure 5). Taken with the pAKT and TUNEL staining, these data show that the AKT inhibitor virtually completely blocks the beneficial effects of FGF7p, making it very likely that the FGF7p urothelial cytoprotection is via AKT signaling. Moreover, these data mimic what we observed with full‐length FGF7, suggesting that the mechanism of the FGF7p cytoprotection against cyclophosphamide is the same as its full‐length parent.

## 4 DISCUSSION

This study builds on our previous work showing that FGF7p, a 19 amino acid derivative of human FGF7, was able to block cyclophosphamide‐induced apoptosis in bladder urothelial intermediate and basal cells as effectively as the full‐length ligand (Narla et al., 2022). Here we determined that the suppression of urothelial apoptosis led to more rapid and higher fidelity repair of urothelium at 28 days post‐injury, similar to the full‐length peptide (Narla, Bushnell, Schaefer, Nouraie, & Bates, 2020). Vehicle 1‐treated mice had ongoing urothelial hyperplasia, incomplete restoration of KRT20 staining, persistent KRT14^+^ basal progenitors, and ongoing proliferation (particularly in KRT14^+^ cells), consistent with ongoing regeneration and incomplete repair; however, FGF7p‐treated and injured mice had urothelium that largely resembled quiescent and uninjured urothelium with a typical number of urothelial cell layers, largely restored KRT20 staining and limited numbers of proliferating and/or KRT14^+^ cells. In addition, we observed foci of ectopic lumenal KRT14^+^ cells in Vehicle 1‐treated mice 28 days after injury, similar to what we observed in injured wild‐type mice in a different study (Narla, Bushnell, Schaefer, Nouraie, Tometich, et al., 2020), which may explain some of the heightened urothelial cancer risks in patients who received cyclophosphamide. Notably, FGF7p treatment completely blocked ectopic lumenal KRT14 expression, which could translate into lowered cancer risk if patients received the peptide.

The current study also strongly supports that FGF7p‐driven suppression of urothelial apoptosis after cyclophosphamide depends on intact AKT signaling, similar to full‐length FGF7 (Narla et al., 2021). Co‐administration of the AKT_i_ (LY294002) largely blocked FGF7p‐driven urothelial pAKT staining and urothelial cytoprotection. We also demonstrated a critical role for AKT in mediating urothelial cytoprotection in that a direct AKT agonist could substitute for full‐length FGF7 in blocking urothelial apoptosis (Narla et al., 2021). Studies in many non‐bladder cells and organ systems have shown that AKT signaling can block apoptosis, often downstream of FGF signaling (Bao et al., 2005; Cai et al., 2018; Chang et al., 2009; Qiu et al., 2010; Song et al., 2005). Our previous studies with full‐length FGF7 support that the anti‐apoptotic effects of AKT activation on cyclophosphamide‐injured urothelium is likely via its actions on BCL2 associated agonist of cell death (BAD) and mammalian target of rapamycin complex 1 (mTORC1) (Narla et al., 2021; Narla, Bushnell, Schaefer, Nouraie, & Bates, 2020). These and/or other AKT targets likely drive the FGF7p cytoprotection in urothelium subjected to cyclophosphamide. Together, it appears that stimulating AKT signaling in the urothelium via FGF7p, full‐length FGF or a direct AKT agonist blocks cyclophosphamide‐induced apoptotic urothelial cell injury.

On balance, the use of FGF7p to mitigate bladder injury from cyclophosphamide has many potential advantages over full‐length FGF7 or direct AKT agonists. One minor disadvantage compared to full‐length FGF7 is that the threshold dose of FGF7p required to drive pAKT expression in the bladder is higher than FGF7 (20 mg/kg vs. 5 mg/kg, respectively) (Narla et al., 2022). The biggest advantage of FGF7p over FGF7 is significant production (and potentially consumer) costs. While full‐length recombinant human (rh) FGF7 costs ~$5200 for 1 mg (R&D Systems, Minneapolis, MN, Cat# 251‐KG‐010), we can synthesize FGF7p in the University of Pittsburgh Peptide Synthesis core for ~$24 for 1 mg (<0.5% of the commercial cost of rhFG7). If FGF7p were mass‐produced, it would lead to more cost savings. Lyophilized FGF7p also has a longer shelf life (5–10 years) than rhFGF7 (1 year‐R&D Systems) and direct synthesis allows for higher purity than recombinant production. While systemic use of FGF7 appears safe, particularly as a “one‐off” therapy, recurrent systemic FGF7 dosing could lead to unwanted side effects. A potential solution to any unwanted side effects could be to directly instill FGF7 transurethrally into the bladder; however, full‐length FGF7 is likely too large to be taken up by urothelial cells (data from a rodent study also indirectly support a non‐trophic effect of local KGF on urothelium [Ulich et al., 1997]). Conversely, the relatively small size of FGF7p might allow it to traverse the urothelial barrier and work if instilled directly into the bladder through the urethra. While small lipophilic AKT agonists (including SC‐79 which we used in our previous study [Narla et al., 2021]) should likely cross the urothelial barrier as well, a concern is that the AKT agonist would likely activate AKT in all tissues, including bladder stromal and vascular tissues; conversely, FGF7p or FGF7 will only drive AKT signaling where their receptor, FGFR2IIIb, is expressed, which in the context of the bladder is only the urothelium. Systemic AKT agonist infusions would also likely activate AKT in tumors, blocking the effectiveness of cyclophosphamide, unlike FGF7p or FGF7, given that lymphoma cells do not express FGFR2IIIb (Orr‐Urtreger et al., 1993). Finally, while beyond the scope of this study, FGF7p might block other types of bladder urothelial injury such as radiation or neuropathic injury or could protect against other types of epithelial injury for which FGF7 is beneficial such as oral, retinal, alveolar, or intestinal epithelial injury (Dorr et al., 2002; Farrell et al., 1998; Farrell et al., 1999; Farrell et al., 2002; Hu et al., 2018; Khan et al., 1997; Takeoka et al., 1997; Wu et al., 1998).

## AUTHOR CONTRIBUTIONS

Sridhar Narla: Study design; conducting experiments; analysis; writing and editing. Lori Rice: Conceptualization; analysis; writing, reviewing, and editing. David Ostrov: Conceptualization; analysis; writing, reviewing, and editing. Daniel Bushnell: Conducting experiments; writing. Joanne Duara: Conducting experiments; writing. Carlton Bates: Conceptualization; supervision; analysis writing, reviewing, editing.

## ETHICS STATEMENT

We used two‐to‐three month old female FVB/NJ mice for all assays (The Jackson Laboratory, Bar Harbor, ME). All of the mouse assays were approved by the University of Pittsburgh Institutional Animal Care and Use Committee in compliance with guidelines from the Association for Assessment and Accreditation of Laboratory Animal Care.
